# The impact of minimally invasive restorative techniques on perception of dental pain among pregnant women: a randomized controlled clinical trial

**DOI:** 10.1186/s12903-021-01432-3

**Published:** 2021-02-18

**Authors:** May M. Adham, Mona K. El Kashlan, Wafaa E. Abdelaziz, Ahmed S. Rashad

**Affiliations:** 1 Department of Pediatric Dentistry and Dental Public Health, Faculty of Dentistry, Alexandria University, Champolion St., Azarita, Alexandria, 21527 Egypt; 2 Department of Economics, Faculty of Commerce, Damanhour University, Damanhour, Egypt

**Keywords:** Chemo-mechanical caries removal, Papacarie-duo, Pregnant women, Atraumatic restorative treatment, Minimal invasive dentistry, Dental pain, Patient satisfaction, Excavation time

## Abstract

**Background:**

The public dental care sector is striving to fulfill the preventive and restorative needs of Egyptians, including pregnant women, who may not receive timely care due to misconceptions about dental treatment during pregnancy. Because of this, they are likely to suffer dental pain, with higher risk of infection affecting their offsprings.

**Aim of the study:**

To compare the effectiveness of chemo-mechanical caries removal using Papacarie-Duo and Atraumatic Restorative Treatment (ART) in reducing dental pain among pregnant women.

**Materials and methods:**

A randomized controlled clinical trial was conducted, in 2019, and included 162 pregnant women visiting family health centers in Alexandria, Egypt, with dental pain due to dental caries not extending to pulp. Patients were randomly assigned to Papacarie-Duo group (n = 82) and ART group (n = 80) after stratification by number of treated surfaces. The outcome variables were reduction in pain assessed using Visual Analogue Scale (VAS), satisfaction with treatment, and time taken for dental caries removal. T test/ Mann Whitney U test were used to compare groups and Freidman test was used to compare change across time.

**Results:**

Pain reduction was significantly greater in the Papacarie-Duo than the ART group (81.55% and 69.43%, *P* = 0.001). Patients in the Papacarie-Duo group were significantly more satisfied with treatment than those in the ART, immediately after treatment (mean = 9.60 and 8.00, *P* =  < 0.01) and after 6 months (mean = 9.63 and 8.16, *P* =  < 0.01). Significantly less excavation time was recorded in the Papacarie-Duo group than in the ART group (mean = 10.38 and 11.56 min, *P* =  < 0.01).

**Conclusion:**

Chemo-mechanical caries removal using Papacarie-Duo is more effective in reducing dental pain, in pregnant women, and is associated with more satisfaction and less excavation time than ART.

*Trial registration*: ID NCT04573608 (https://clinicaltrials.gov/); 5/10/2020, retrospective registration.

## Background

Physical and hormonal changes affect pregnant women in many ways and the oral cavity is no exception. There are many common oral problems during this stage such as pregnancy gingivitis, benign gingival lesions, tooth mobility, tooth erosion, periodontitis and dental caries [1]. Dental caries is a biofilm-mediated, diet modulated, multifactorial, non-communicable, dynamic disease resulting in net mineral loss of dental hard tissues. It is determined by biological, behavioral, psychosocial, and environmental factors. As a consequence of this process, a caries lesion develops [2, 3]. The increased risk for developing dental caries during this stage can occur due to several factors; the oral cavity is characterized by an acidic environment due to an inflammatory response brought about by vomiting, gastric-reflux and changes in salivary composition. This is further complicated by the high sugar cravings that are common during this period as well as the limited attention to oral health [4]. Thus, the presence of untreated dental caries reflects unmet treatment need rather than higher incidence of disease during this period. Studies showed that if dental caries is left untreated, may in some cases result in further inflammatory complications which could influence pregnancy outcomes like preterm birth and/or low birth weight [5–7].

Despite these consequences, pregnant women, sometimes, do not seek dental treatment, not only due to misconceptions about the safety of dental procedures during pregnancy but also because some dentists may be reluctant to provide dental treatment [8].

In Rio de Janeiro, Brazil, 39% of the participating pregnant women experienced dental pain, which affected their daily normal activities [9]. Whereas, in South Brazil, prevalence of dental pain was found to be 54.9% [10]. Unlike other types of pain experienced during pregnancy, pain due to dental caries can be avoided by early treatment instead of analgesics that may affect the infant’s health [11].

There has been an increased interest in the use of minimally invasive restorative procedures as an alternative to traditional rotary techniques of dental caries removal to overcome its various disadvantages such as vibration, pressure and heat generation. These minimally invasive methods include chemo-mechanical agents and atraumatic restorative treatment (ART) [12].

The ART technique involves dental caries removal using hand instruments, followed by cavity filling using glass ionomer. There is no need for anesthesia and pain is reduced to minimum. This approach was originally developed for use outside the dental clinic, in remote areas with no electricity [13, 14]. It can also be used for children, elderly population, patients with special needs and those who experience dental anxiety [15]. Shenoy et al. [16] assessed pregnant women’s response to ART, in primary health care centers, and reported a high level of satisfaction with the procedure where none of the participants felt pain during treatment.

Chemo-mechanical caries removal, on the other hand, involves the use of chemical solutions which soften dental caries and facilitate its removal using hand instruments. There are two categories of chemo-mechanical caries removal agents: sodium hypochlorite (NaOCl) such as Carisolv, and enzyme-based agents where Papacarie is considered its most common agent [17]. Papacarie-Duo, the newer version of the product, was developed in 2011, and has greater viscosity allowing more precise application, longer shelf life with no need to be refrigerated.

Most studies involving oral health of pregnant women focused on periodontal conditions [18–21], while few studies were concerned with pain caused by dental caries [10, 16]. Furthermore, several studies compared conventional drilling and chemo-mechanical treatments [22–25]. However, only few studies compared ART and chemo-mechanical treatment, and those were mostly conducted in children. Only one pilot study compared chemo-mechanical treatment using Carisolv and ART, among 50 pregnant women, in order to assess the longevity of glass ionomer restoration [26]. Given the advantage of Papacarie-Duo over Carisolv in relation to lower cost and time taken in dental caries excavation [17], it may have added advantage in countries with limited resources and facilities, like Egypt. Therefore, if its effectiveness is proven, Papacarie-Duo can be a feasible method to treat dental caries and reduce dental pain among pregnant women during routine prenatal care in family health centers.

The aim of the study was, thus, to compare the effectiveness of two minimally invasive caries removal techniques: chemo-mechanical (Papacarie-Duo) and ART, in pregnant women, regarding pain reduction after one month and six months, their satisfaction with the treatment procedure and time taken for dental caries removal. The null hypothesis was that there would be no statistically significant difference between the two modalities in pain reduction.

## Methods

### Study design

A randomized, two parallel arms, controlled clinical trial was conducted among pregnant women attending family health centers for their routine prenatal care in Alexandria, Egypt, from January to October 2019.

The study was conducted in accordance with the CONSORT guidelines[27] and Helsinki declaration; and it was approved by the Research Ethics Committee, Faculty of Dentistry, Alexandria University (IRB 00010556-IORG 0008839). Permission to access the family health facilities was obtained from the Health Directorate of Alexandria. Signed written informed consents were obtained from patients after explaining the aim of the study, risks, benefits and confirming confidentiality of their response as well as their right to withdraw at any time. The trial was registered at clinicaltrial.gov (NCT04573608) on 5/10/2020.

### Participants

Pregnant women were eligible to participate if they were in the first or second trimester, had at least mild dental pain as identified by a score of at least 5 mm on a Visual Analogue Scale (VAS) 100-mm-long [28], and with at least one carious lesion involving dentine clinically classified as a shallow or medium cavity. This cavity should be accessible to hand instruments (International Caries Detection and Assessment System (ICDAS) score = 5 or 6) [29]). Pregnant women with acute pulpitis, swelling or fistula as well as uncooperative patients, those having severe gingivitis (Gingival Index (GI) score = 3 [30]), those who are unable to read and/or write and those who refused to participate were excluded from the study.

Sample size was based on assuming a 5% alpha error, 20% beta error and reported percentage of patients with pain after using chemo-mechanical caries removal = 68% and 35% after ART [31]. The minimum required number of patients was estimated to be 33 per group [32]. This was increased to 40 to make up for loss to follow up. Pain and effect of the two treatment modalities was assumed to be affected by the extent of dental caries and; hence, stratified by the number of surfaces affected by dental caries into single and multi-surface lesions, creating two strata per group. The number of participants, therefore, per group was 80 with a total of 160 participants.

### Randomization

A computer-generated list of random numbers was used to randomly assign patients into one of the two study groups in a ratio 1:1 in blocks of four [33]. The allocation sequence was concealed from the researcher administering the intervention in sequentially numbered, opaque, sealed envelopes [34]. Blinding of participants was not possible due to the difference between the two techniques.

### Interventions

#### Group I: Papacarie-Duo

Papacarie was introduced into the cavity using the applicator and left for 40 s and a blunt excavator was used to remove the softened dentin. The remaining gel was removed using a cotton pellet. When there was no change in gel color, the cavity was considered caries free [23]. The cavity was then filled with high viscosity glass ionomer cement (GIC) in an encapsulated form (Riva Self-Cure, SDI Limited, Bayswater, VIC, Australia). A mechanical mixer was used to mix the capsule for 10 s, the capsule was placed into the applicator to apply the GIC into the cavity. For occluso-proximal cavities, a matrix strip with a wooden wedge was used to provide the appropriate contour of the restoration. A gloved finger was used to apply pressure on the GIC for one minute and occlusion was checked and excess material was removed [35].

#### Group II: ART

The tooth was cleaned with a wet cotton pellet to remove debris and plaque, dental caries was then removed using sharp spoon excavators (Darby-Perry #220/221, #17 DE, Hu-Friedy, Chicago, USA), followed by cleaning the cavity using a small wet cotton pellet and finally dried with a dry cotton pellet. The cavity was considered caries-free when a leather-hard texture was reached and the excavator did not stick anymore [14]. The GIC was used to restore the cavity using the same technique described for the other group [33].

### Outcomes assessment

- Difference in pain was measured using Visual Analogue Scale (VAS) at baseline, after one month and a six months period. The scale is represented by a 100-mm-long horizontal line labeled “no pain” at one end and “worst pain” at the other end. Participants were asked to mark the place on the line representing their level of pain [28].
- Satisfaction with treatment was assessed using two questions: the first question was immediately following treatment: “Was the treatment carried out according to your expectations?” The second question was after 6 months” “Has the treatment solved the problem of your teeth?” Each question was answered on a 10-point scale, with lower values indicating a negative perspective and higher values indicating a positive experience [36].
- Time to remove dental caries was recorded using a stop watch from the start of excavation up to complete dental caries removal [37]
- To control for potential confounders, information about age, education level, pregnancy stage, brushing frequency and last dental visit as well as clinical oral health indicators like number of decayed teeth, gingival index and plaque index were collected using the Arabic version of the WHO oral health assessment questionnaire for adults which was previously validated [38]. Clinical examination assessed the number of decayed teeth based on WHO criteria [39]. The amount of dental plaque and the gingival condition were both assessed using Silness and Loe [40] and Loe and Silness criteria [30].

### Statistical analysis

SPSS (Version 24.0, IBM Corp., Armonk, N.Y., USA) was used for data analysis. Quantitative variables were checked for normality using Shapiro Wilks tests, histograms and QQ plots. Descriptive statistics were displayed as frequencies and percentages for categorical variables and means and standard deviations for quantitative variables. Independent t test or Mann Whitney U test (depending on normality) was used to assess differences in quantitative variables: age, number of decayed teeth, gingival index, plaque index, pain and satisfaction scores. Chi square was used for categorical variables: education level, brushing frequency and last dental visit. Friedman test used to assess change in VAS across time in each group. An intention-to-treat analysis was performed by assigning the worst pain and satisfaction scores to those lost to follow up using worst case scenario analysis. Significance level was set at *P* < 0.05.

## Results

**Fig. 1:**
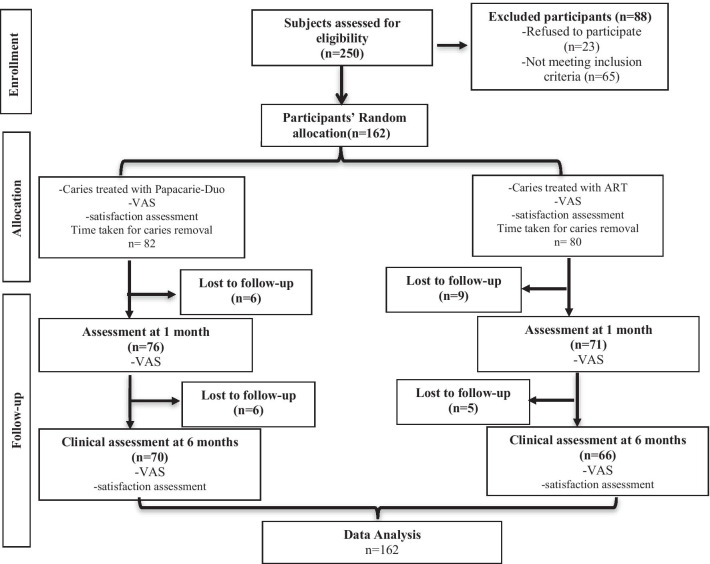
Flow chart showing flow of patients at various stages of the study

Among 250 pregnant women assessed for eligibility, 162 participants fulfilled the inclusion criteria and were randomly allocated to Papacarie-Duo group (n = 82) or ART group (n = 80) (Fig. 1). The dropout rate after six months was 14.6% and 17.5%, respectively.

Table 1 presents general characteristics of the study sample, mean age (SD) of pregnant women in the Papacarie-Duo and ART groups were: 27.1 (2.82) and 26.3 (3.18). The majority of pregnant women received secondary education (58.5% and 63.8% respectively), were in the second trimester (64.6% and 73.8% respectively), brushed at least once per day (53.7% and 42.5% respectively) and slightly more pregnant women in the ART group than those in the Papacarie-Duo group visited the dentist within the last year (57.5% 47.6% respectively).

**Table 1:** Comparison of pregnant women in the study groups regarding personal background, oral health practices, and oral health indicators

	Papacarie-Duo n = 82	ART n = 80
Age mean (SD)	27.1 (2.82)	26.3 (3.18)
Education n (%)		
Primary education	12 (14.6%)	9 (11.3%)
Secondary education	48 (58.5%)	51 (63.8%)
Higher education	22 (26.8%)	20 (25%)
Brushing frequency n (%)		
Less than once per day	38 (46.3%)	46 (57.5%)
At least once per day	44 (53.7%)	34 (42.5%)
Last Dental visit n (%)		
Within the last year	39 (47.6%)	46 (57.5%)
More than 1 year, less than 5 years	38 (46.3%)	32 (40%)
More than 5 years	5 (6.1%)	2 (2.5%)
Trimester n (%)		
First trimester n (%)	29 (35.4%)	21 (26.3%)
Second trimester n (%)	53 (64.6%)	59 (73.8%)
Diet		
Sweets/candies		
At least daily	45 (54.9%)	48 (60%)
Less than daily	37 (45.1%)	32 (40%)
Tea with sugar		
At least daily	61 (74.4%)	54 (67.5%)
Less than daily	21 (25.6%)	26 (32.5%)
Fruits		
At least daily	30 (36.6%)	29 (36.3%)
Less than daily	52 (63.4%)	51 (63.8%)
No. of decayed teeth mean (SD)	2.91 (0.86)	2.71 (0.92)
Gingival Index mean (SD)	1.54 (0.32)	1.43 (0.29)
Plaque Index mean (SD)	1.69 (0.34)	1.65 (0.29)
Type of cavities n (%)		
One-surface cavities	45 (54.9%)	41 (51.3%)
Multi-surfaces cavities	37 (45.1%)	39 (48.8%)

*Statistically significant at *P* < 0.05

Regarding the consumption of sugar at least once per day, tea with sugar was the most common source of sugar reported by 74.4% of women in the Papacarie-Duo group and 67.5% of those in the ART, followed by sweets/candies: (54.9% and 60% respectively). Consumption of fresh fruits at least once daily was less common: (36.6% and 36.3% respectively).

There were no differences between pregnant women in the Papacarie-Duo and ART groups regarding number of decayed teeth: mean (SD) = 2.91 (0.86) and 2.71 (0.92), plaque accumulation: mean (SD) = 1.69 (0.34) and 1.65 (0.29), or extent of dental caries: single surface = 54.9% and 51.3%. Women in the Papacarie-Duo had more gingivitis than those in the ART group: mean (SD) = 1.54 (0.32) and 1.43 (0.29).

Table 2 shows no significance difference in pain perception between pregnant women in the Papacarie-Duo and ART groups at baseline: mean (SD) = 22.38 (11.53) and 23.75 (11.73), *P* = 0.454, including those with single surface cavities: 18.33 (11.28) versus 18.29 (10.22), *P* = 0.987 and those with multiple surfaces cavities: 27.3 (9.9) versus 29.49 (10.5), *P* = 0.353.

**Table 2:** Difference in pain scores between groups and across time

	Papacarie-Duo	ART	*P* value
	mean (SD)	mean (SD)	
One surface cavities (n = 86)			
Baseline	18.33 (11.28)^a^	18.29 (10.22)^a^	0.986
1 month	3.67 (6.16)^b^	5.85 (7.15)^b^	0.100
6 months	2.33 (4.88)^b^	4.88 (6.66)^b^	0.044*
*P* value	< 0.001*	< 0.001*	
Percent reduction	89.53 (23.78)	79.41 (29.49)	0.084
Multi- surface cavities (n = 76)			
Baseline	27.3 (9.9)^a^	29.49 (10.5)^a^	0.353
1 month	8.11 (7.11)^b^	13.46 (8.75)^b^	0.008*
6 months	7.43 (7.32)^b^	12.82 (9.23)^b^	0.012*
*P* value	< 0.001*	< 0.001*	
Percent reduction	72.07 (31.67)	58.93 (27.91)	0.049*
All cavities (n = 162)			
Baseline	22.38 (11.53)^a^	23.75 (11.73)^a^	0.454
1 month	5.67 (6.93)^b^	9.56 (8.79)^b^	0.003*
6 months	4.63 (6.56)^b^	8.75 (8.9)^b^	0.002*
*P* value	< 0.001*	< 0.001*	
Percent reduction	81.55 (28.84)	69.43 (30.35)	0.01*

*Statistically significant at *P* ≤ 0.05

^a,b^Different letters denote significant difference between time points within each group

At one month, women in the Papacarie-duo group had significantly less pain than women in the ART group regarding all cavities: mean (SD) = 5.67 (6.93) and 9.56 (8.79), *P* = 0.003 and in multiple surfaces cavities: mean (SD) = 8.11 (7.11) and 13.46 (8.75), *P* = 0.008. No significant difference was noted between groups among women with single surface cavities: 3.67 (6.16) and 5.85 (7.15), *P* = 0.100.

After 6 months, women in the Papacarie-duo group had significantly less pain than those in the ART group whether in the whole sample: mean (SD) = 4.63 (6.56) and 8.75 (8.9), *P* = 0.002, in those with single surface cavities: 2.33 (4.88) and 4.88 (6.66), *P* = 0.044 or in those with multiple surface cavities: 7.43 (7.32) and 12.82 (9.23), *P* = 0.012.

The reduction in pain from baseline to one and 6 months was statistically significant in both groups (*P* =  < 0.001). The difference in the percent reduction from baseline to 6 months between the groups was statistically significant for the whole sample: 81.55 (28.84) and 69.43 (30.35), *P* = 0.01, for women with multiple surface cavities: 72.07 (31.67) and 58.93 (27.9), *P* = 0.04 but not among women with single surface cavities: 89.53 (23.78) and 79.41 (29.49), *P* = 0.084.

The mean (SD) satisfaction scores immediately after treatment and after 6 months were significantly higher in the Papacarie-Duo: 9.6 (0.68) and 9.63 (0.59) than in the ART group: 8 (0.87) and 8.16 (0.89), *P* < 0.001, whether among women with single surface cavities (Papacarie-Duo: 9.84 (0.42) and 9.84 (0.37) versus ART: 8.32 (0.93) and 8.27 (0.89), *P* < 0.001) or with multiple surface cavities: Papacarie-Duo: 9.3 (0.81) and 9.38 (0.72) versus ART: 7.67 (0.66) and 8.05 (0.89), *P* < 0.001. The change in satisfaction across time in the two groups was not statistically significant for the whole sample (*P* = 0.320 and 0.096 for Papacarie-Duo and ART, respectively) and for women with single surface cavities (*P* = 1.00 and 0.728, respectively). In women with multiple surfaces cavities, there was no significant difference in the Papacarie-Duo (*P* = 0.183), while in the ART group, the satisfaction after 6 months increased significantly (*P* = 0.004), Table 3.

**Table 3:** Difference in the level of satisfaction between the two study groups

	Papacarie-Duo	ART	*P* value
	mean (SD)	mean (SD)	
One surface cavities (n = 86)			
Satisfaction after treatment	9.84 (0.42)	8.32 (0.93)	< 0.001*
Satisfaction after 6 months	9.84 (0.37)	8.27 (0.89)	< 0.001*
*P* value	1.00	0.728	
Multi- surface cavities (n = 76)			
Satisfaction after treatment	9.30 (0.81)	7.67 (0.66)	< 0.001*
Satisfaction after 6 months	9.38 (0.72)	8.05 (0.89)	< 0.001*
*P* value	0.183	0.004*	
All cavities (n = 162)			
Satisfaction after treatment	9.60 (0.68)	8.00 (0.87)	< 0.001*
Satisfaction after 6 months	9.63 (0.59)	8.16 (0.89)	< 0.001*
* P* value	0.320	0.096	

*Statistically significant at *P* ≤ 0.05

A significant negative moderate correlation was found between pain and satisfaction at 6 months (r = − 0.339, *P* =  < 0.001) indicating that patients who reported less pain were more satisfied with the treatment provided.

The mean (SD) time in minutes taken for dental caries removal was significantly less in the Papacarie-Duo-duo: 10.38 (1.94) for all cavities, 9.36 (1.8) for one surface cavities and 11.62 (1.28) for multiple surfaces cavities than in the ART group: 11.56 (1.62) for all cavities, 10.71 (1.37) for one surface cavities and 12.46 (1.37) for multiple surfaces cavities, *P* =  < 0.001, Table 4.

**Table 4:** Comparison of time in minutes for dental caries removal in Papacarie-Duo and ART groups

	Papacarie-Duo	ART	*P*
	Mean (SD)	Mean (SD)	
One surface (n = 86)	9.36 (1.8)	10.71 (1.37)	< 0.001*
Multi-surfaces (n = 76)	11.62 (1.28)	12.46 (1.37)	0.007*
All cavities (n = 162)	10.38 (1.94)	11.56 (1.62)	< 0.001*

*Statistically significant at *P* ≤ 0.05

## Discussion

Papacarie-Duo was associated with significantly greater pain reduction, higher satisfaction and less time of dental caries excavation than ART in pregnant women. The greater effectiveness of Papacarie-Duo was more observed in multi-surface cavities when pain and excavation time were considered whereas greater satisfaction compared to ART was reported after 6 months in one surface cavities. The null hypothesis of the study can, therefore, be rejected. The current findings have implications for the dental care of women who suffer from dental pain during pregnancy and are reluctant to seek conventional treatment for fear that it may harm their babies. Even if dental caries extends to more than one surface, it can still be restored with Papacarie-Duo if adequate access for the instruments can be ensured. The duration of the trial shows that this treatment can adequately last throughout the period of pregnancy after which other types of treatment can be provided if needed. Further studies with longer follow up and with economic evaluation are needed to inform policy decision makers about the use of Papacarie-Duo as an efficient restorative modality in low-resource settings.

Pain reduction was greater in multi-surfaces cavities treated with Papacarie-Duo than ART whereas there was no difference in one surface cavities. This may be attributed to the components of the gel that contains papain enzyme which has an anti-inflammatory, bactericidal and bacteriostatic action. Papacarie-Duo removes carious tissues without affecting the sound collagen fibers in the affected normal dentin, and as a result, less instrumentation is required with less pain generated during excavation [41]. This finding agrees with Ericson et al. [42] who showed that the chemo-mechanical approach is effective and more comfortable for patients compared to ART where operators sometimes tend to “dig” excavators into the hard carious dentine breaking off hard dentine pieces not only the leathery-firm dentin, thereby opening more dentine tubules which induces pain [43].

The present study is the first to evaluate the effect of two minimally invasive restorative techniques (Papacarie and ART) on pain reduction and satisfaction among adults. Only one study by Barata et al. [26] compared ART to chemo-mechanical caries removal using Carisolv in pregnant women, however, they assessed restoration longevity not pain and found similar clinical performance of the two methods after 12 months. Another study concluded that both Carisolv and Papacarie were clinically efficient in dental caries removal among those aged 20–40 years, while Papacarie was better regarding time taken and the volume of excavated carious tissues [41].

In the present study, there was a significant increase in satisfaction among patients with multiple surfaces cavities treated by ART, after 6 months, but not among those treated with Papacarie-Duo. This may be attributed to the fact that some patients felt relatively mild pain during excavation in larger cavities and thus longer time was taken to completely remove dental caries than in single surface cavities. This led to initially lower levels of satisfaction with subsequent potential for improved satisfaction as time passed and dental pain resolved. Although direct comparison is not possible, these findings were in line with Anegundi et al. [44] who reported that 60% of participants preferred Papacarie compared to 36.7% preferring traditional methods and Goyal et al. [45] who reported that 80% preferred Papacarie caries removal over the conventional drilling. Furthermore, the study conducted by Kumar et al. [41] on adult population comparing Papacarie and Carisolv revealed that patients, in both groups, were satisfied with the treatment provided as no pain was reported during dental caries removal. Patients’ satisfaction may have been partially related to the dental pain reported at 6 months. This is supported by the significant negative correlation between pain and satisfaction, reported in the current study, at 6 months.

The present study revealed significantly shorter time of excavation in the Papacarie-duo group than in the ART, in both single and multiple surface cavities. This is because Papacarie-Duo softens dental caries so that it is easily removed. This is comparable to other studies of permanent dentition, in adults, where the time for excavation in the present study was slightly less in the Papacarie-Duo group as compared to that reported by Kumar et al. [41] and less than that recorded for Carisolv by Barata et al. [26].

One of the limitations of the study is that blinding could not be applied due to the differences between the two techniques. Also, patients who received Papacarie-Duo may have over-reported their satisfaction because of their attraction to the new material and some patients had difficulty translating pain to a linear scale. There was a slight difference between groups in the severity of gingivitis. However, both groups were in the mild to moderate level of gingival inflammation and thus, this minor difference is unlikely to affect the study conclusions. Also, in the Papacarie-Duo group, the cavity was considered caries free when the gel stayed clear. On the other hand, in the ART group, excavating till hard dentin was reached might have been responsible for greater pain felt with less pain reduction reported [3, 43]. Future studies assessing these different non-invasive methods should consider having similar criteria of declaring the cavity caries free. In addition, chemomechanical caries removal can be used only if cavities are open so that an excavator can be used which limits the generalizability of findings only to those with this special form of cavities.

Papacarie-Duo is available at low cost (73$ per tube which may be used for 25 applications) providing a cost reduction of 42% compared to traditional drilling method [46]. Although no data are available about the market availability of Papacarie per country, research shows that studies about Papacarie are conducted in South America, Europe, India, Pakistan, United States and Egypt [17].

This study was performed in public healthcare centers which mainly serve the disadvantaged communities. Hence, this new low-cost line of treatment is suggested for use in remote areas, deprived from proper facilities and equipment. In addition, this study aids in filling the knowledge gap by providing evidence about the effectiveness of minimally invasive caries treatment in adults which makes possible the translation of the results of scientific research to clinical practice. Future studies, based on action research, are needed to evaluate the feasibility and cost-effectiveness of integrating minimally invasive caries removal into comprehensive dental care programs for pregnant women.

## Conclusion

Chemo-mechanical caries removal using Papacarie-Duo is more effective in reducing dental pain, in pregnant women, and is associated with more satisfaction and less excavation time than ART which offers an alternative low-cost method for treatment of dental caries among women who suffer from dental pain during pregnancy but and are reluctant to seek conventional treatment.

## Data Availability

The dataset used in this research is available at synapse.org under the title: The impact of minimally invasive restorative techniques on perception of dental pain among pregnant women. Synapse ID: syn23521994. User name: @may.adham.

## Abbreviations

ART: Atraumatic Restorative Treatment
VAS: Visual Analogue Scale
ICDAS: International Caries Detection and Assessment System
GI: Gingival Index
GIC: Glass Ionomer Cement
SPSS: Statistical Package for Social Sciences
